# Association of physical activity level during pregnancy with preterm birth and infant adiposity: longitudinal analysis from the MAASTHI cohort, India

**DOI:** 10.1007/s11332-025-01409-7

**Published:** 2025-05-08

**Authors:** Yamuna Ana, Floor A. van den Brand, Onno C. P. van Schayck, Giridhara R. Babu

**Affiliations:** 1 Department of Family Medicine, Care and Public Health Research Institute (CAPHRI), Maastricht University, Maastricht, Limburg The Netherlands; 2 Indian Institute of Public Health-Bengaluru, Public Health Foundation of India (PHFI), Bengaluru, India; 3 Department of Family Medicine, Care and Public Health Research Institute, (CAPHRI), Maastricht University, Maastricht, Limburg The Netherlands; 4 Department of Population Medicine, College of Medicine, QU Health, Qatar University, P.O. Box: 2713, Doha, Qatar

**Keywords:** Anthropometry, Birth cohort, Delivery gestational age, Obesity, Physical activity

## Abstract

**Background:**

Physical activity during pregnancy is thought to influence birth outcomes, but its association with it is not fully understood. We aimed to examine the association of sedentary behavior during pregnancy with preterm birth and infant adiposity measured at birth, 14 weeks, and one year of age.

**Methods:**

In this cohort study, physical activity during pregnancy was assessed using the physical activity questionnaire. Gestational age at birth was determined soon after delivery and the infants were followed up until one year of age. Infant anthropometric measurements such as weight, length, crown–rump length, mid-upper arm circumference, waist and hip circumference, and skinfold thickness were assessed at each follow-up visit. Multivariable linear and logistic regression models were used to assess the association of sedentary behavior during pregnancy with preterm birth and infant adiposity, after adjusting for confounders.

**Results:**

Among the 1315 pregnant women who participated in the study, 7.1% had sedentary behavior. Sedentary behavior during pregnancy was significantly associated with a higher incidence of preterm birth (OR = 1.43, *p* = 0.041). Sedentary behavior during pregnancy showed a significant association with adiposity in children as measured through hip circumference (OR = 2.53, *p* = 0.007) at the 14-week follow-up and the sum of skinfold thickness (OR = 1.53, *p* = 0.024) at one-year follow-up.

**Conclusion:**

This study highlights a significant association between sedentary behavior during pregnancy and preterm birth, markers of adiposity in children from birth to one year of age. These findings underscore the importance of maintaining moderate physical activity during pregnancy.

## Introduction

Pregnancy is an important time for the health of both the mother and the fetus. Health behaviors of the mother during this period can have a significant impact on the short- and long-term outcomes of the child. One such practice is physical activity (PA) during pregnancy, which is increasingly recognized as a crucial factor in promoting newborn health by reducing developmental problems in infants [1], improving neonatal cortical development, [2] and reducing children’s fat mass [3]. Pregnant and postpartum women are recommended to perform some form of PA for at least 150 min per week [4]. Although PA plays an important role in maternal and infant health, pregnant women tend to perform mostly sedentary behavior [5]. In Karnataka, India, the majority of the pregnant women had a recommended level of physical activity in rural areas [6]. In addition, pregnant women have a poor understanding of the recommended level of PA which is required during pregnancy [7].

Moderate physical activity has beneficial effects for pregnant women who were sedentary during the pre-pregnancy period, [8] and it is essential for better health outcomes among pregnant women and their newborns [9]. There is also evidence that the timing of PA in pregnancy may be important. It is safe for pregnant women to engage in some form of exercise as it can benefit both the mother and the newborn without harming their health [10]. However, according to guidelines in several countries strenuous activity and certain types of exercise that involve uneven strain during stretching, excessive body heat, excessive joint stress, and sports involving falls should be avoided during pregnancy to prevent adverse outcomes [9]. It may be important to avoid long working hours, prolonged standing, and heavy physical work, particularly late in pregnancy [11]. PA during mid-pregnancy was positively associated with maternal cardiometabolic health and the absence of neonatal adiposity [12]. Physically active pregnant women are less likely to have adverse maternal health outcomes such as cesarean delivery, [13] instrumental delivery, [14] Gestational Diabetes Mellitus (GDM) [15], hypertension, obesity, [16] and maternal depression [17]. Existing evidence on the association of maternal PA during pregnancy with child anthropometric characteristics in the longer term shows mixed findings. Some studies have shown significant inverse associations with child health outcomes such as preterm birth and infant adiposity [13] large for gestational age infants, [18] and lower infant fat mass [3]. A few studies showed no significant outcomes [19, 20]. Systematic review and meta-analysis found that higher level of physical activity is associated with reduced risk of preterm birth [21], alleviating the high prevalence of childhood obesity [22]. Sedentary behavior is associated with oxidative stress, and elevated inflammatory markers, which triggers uterine contraction and increases the risk of preterm births [23]. It also results in impaired placental function, hormonal imbalance, and insulin resistance potentially leading to early labor [24]. Maternal sedentary behavior is connected to excessive gestational weight gain and higher maternal glucose levels, exposing the fetus to hyperglycemia and excess nutrients. This leads to fetal overnutrition, programming the infant’s metabolism toward higher fat storage after birth [25]. As these children grow older, overweight or obesity may persist [26]. In India, overall 3.4% of children under five years of age are overweight, with 4.2% from urban and 3.2% from rural areas [27]. Therefore, it is important to understand the causes behind it and, more importantly, assess maternal characteristics such as PA.

As the evidence suggests mixed findings regarding the association of physical activity level with preterm delivery and infant adiposity, evidence gathered from this study could fill the knowledge gap in the transgenerational outcome of pregnancy PA. Therefore, there is a need for more robust evidence to clarify whether maternal PA has a protective, neutral, or adverse effect on these outcomes. While short-term effects of pregnancy PA on birth outcomes are studied, the long-term impact on offspring health, such as infant adiposity, remains unclear. Addressing these gaps through well-designed longitudinal studies could provide clearer insights into the role of pregnancy PA in shaping offspring health across generations. Our study aims to examine the association of PA levels during pregnancy on preterm birth and anthropometric markers in children. We hypothesize that there is an association between sedentary behavior among pregnant women and preterm birth as well as infant adiposity. Our primary outcome is preterm birth, and secondary outcomes are infant adiposity measured through anthropometric characteristics of the child at birth, 14 weeks of age, and one year of age.

## Methods

### Study design and setting

This study is nested within a longitudinal cohort study entitled “Maternal antecedents of adiposity and studying the transgenerational role of hyperglycemia and Insulin” (MAASTHI) [28, 29]. This study recruited pregnant women during the gestational age of 14–36 weeks, after obtaining written informed consent. Inclusion criteria include all pregnant women between the age of 18–45 years, who are less than 36 weeks of gestation, planned to deliver in the study hospital, and who reside in the study area. The exclusion criteria included history of diabetes, Hepatitis B infection, and human immunodeficiency virus (HIV) positivity. The study duration was from 2016 to 2021.

Assuming a 7% incidence of adverse infant health outcome among mothers who engage in moderate physical activity during pregnancy, [30] and a relative risk of 1.8 for women with sedentary lifestyle, the estimated sample size required to achieve 80% power for detecting a difference at a 95% confidence level is 878. Accounting for a potential follow-up loss of up to 50%, the adjusted sample size for the study increased to 1317. At the baseline of the cohort, we collected demographic information, socioeconomic characteristics, obstetric history, behavioral factors, depressive symptoms, and social support in addition to measuring blood pressure, GDM status, and anthropometric characteristics such as weight, height, mid-upper arm circumference (MUAC), hip circumference, waist circumference, biceps, triceps, and subscapular skinfold thickness. Within 72 h after delivery, we collected information on the gender of the infant, calculated gestational age at delivery, and measured anthropometry parameters. At the 14-week age and the one-year age of the child, anthropometric measurements were conducted. We collected data using a custom application hosted on Android tablets. To ensure the security and accuracy of the collected data, we implemented strict measures such as real-time monitoring of the data and an audit trail of all data entries made in the application.

### Physical activity assessment

After recruiting the pregnant women, we assessed their PA using a validated physical activity questionnaire [31]. The questionnaire captures information on exercise, hobbies, household chores, sedentary activities, and other everyday activities performed in the past month. Frequencies of the activity were determined using categories of “daily,” “once a week,” “2–4 times a week,” “5–6 times a week,” “once a month,” and “2–3 times a month.”

The duration of each activity was recorded in minutes per day. PAL was measured from the average 24 h Total energy expenditure (TEE) and Basal Metabolic Rate (BMR). To classify the level of PA, we used Physical Activity Level (PAL) cut-offs. Participants were classified as having a sedentary behavior or light activity if their PAL value was 1.40–1.69, moderately active if their PAL value was 1.70–1.99, and vigorously active if their PAL value was 2.00–2.40 [32]. The PA questionnaire was previously validated for epidemiological studies in urban middle class Indians [33]. This questionnaire has been used among pregnant women in the study setting [34]. This questionnaire was assessed for its repeatability and validity. The evaluations of physical activity exhibited a high degree of repeatability (*r* = 0.86, *p* < 0.01). To determine relative validity, energy intake was compared, utilizing repeated 24 h dietary recalls, and energy expenditure was assessed through the physical activity questionnaire, over the same duration. The correlation observed between these two measures was *r* = 0.33 (*p* < 0.05) [33].

### Outcome assessment

#### Primary outcome

The gestational age at birth was categorized based on the World Health Organization (WHO) standards, and if the gestational age at birth was less than 37 weeks, it was defined as preterm birth [35].

#### Secondary outcomes

This study assessed infant adiposity measured at the child’s birth, 14 weeks of age, and at one year of age as secondary outcomes. Adiposity was measured using anthropometry parameters. The trained research staff recorded anthropometric measurements of the newborn within 72 h after delivery, at 14-week follow-up, and at the one-year age of the child. Anthropometric parameters such as the child’s weight were recorded using a weighing scale (SECA 354 weighing scale), and length and the crown–rump length (CRL) were measured with an infantometer (SECA 417 Infantometer). Chest circumference, waist circumference, hip circumference, and MUAC were measured using measuring tape (Chasmors body circumference tape). Skinfold thickness, such as biceps, triceps, and subscapular, was measured using the Caliper (Holtain, UK). The sum of skinfold thickness (SSFT) was calculated by adding up these three skinfold thicknesses. The research staff were periodically tested and certified for anthropometry assessment from St. John’s Research Institute, Bengaluru. We measured weight in kilograms, and readings were taken to the nearest 0.5 g, length in centimeters, circumferences in centimeters, and skinfold thickness in millimeters. We obtained three readings for each measurement. As there is no defined criteria or cut-off to define under-five children obesity measured through anthropometric parameters, and therefore, studies have highlighted the need for research assessing the health outcomes associated with different cut-off points. During childhood and adolescence, any cut-off for anthropometric measurements may represent varying levels of risk depending on age and stage of development due to rapid growth and physiological changes. Evidence suggests that revisions are required for the recommended cut-offs for classifying overweight and obesity [36].The 85 th and 95 th percentile cut-off points for identifying overweight and obesity in children are arbitrary and not associated with health risks related to obesity. Therefore, it is harder to determine anthropometry cut-off points in children as they experience fewer obesity-related illnesses than adults [37]. Studies have also used the 90 th percentile cut-off to define obesity, and therefore, we used both continuous measure and the 90 th percentile cut-off during (logistic) analysis. Adiposity was defined if the distribution was found to be more than the 90 th percentile for anthropometry parameters in logistic regression analysis. Therefore, we presented both continuous and categorized outcomes in assessing association. The ponderal index (PI), which is a measure of the physical growth of infants, is calculated with the equation PI = Weight (GM)/Length (CM)^3^ × 100. Children with an index < 2.0 were classified as having a low ponderal index, 2–2.5 were with normal PI, and > 2.5 were with high PI [38]. Calibration of the anthropometric equipment was performed every month using prescribed guidelines.

### Covariates

This study has assessed several covariates since the proposed objective of our study was influenced by maternal socio-demographic, obstetrical, and physiological factors and infant characteristics. Considering a priori evidence, covariates were included and demographic details such as the age of the pregnant women, education, income, and socioeconomic status were collected during the time of the recruitment. The socioeconomic status was categorized based on education, occupation, and income using the recent version of the Kuppuswamy scale [39]. Details regarding the parity of the pregnant women were collected from the medical records of the pregnant women and verified during the interview. Prenatal depressive symptoms were measured using the Edinburgh Postnatal Depression Scale (EPDS) during the recruitment phase of pregnancy [40]. These questionnaires were validated for use in India [41]. It has ten simple questions, each with four possible responses. Pregnant women were asked to identify the closest response regarding how they felt over the past week. Based on the recorded responses, scores were calculated according to the standard instructions. The total score for EPDS ranged from 0 to 30, and as per the validated norms for EPDS, we considered that women with scores above 13 had symptoms suggestive of depression [42]. We measured social support using a validated social support questionnaire, and median cut-offs were considered for categorizing it into poor and good social support scores [43].

We conducted an Oral Glucose Tolerance Test (OGTT) to diagnose GDM from 24 to 36 weeks of gestational age. We followed the WHO criteria for the classification of GDM [44]. To measure MUAC in pregnant women, the measurer palpated the shoulder and elbow, marked the midpoint, wrapped a horizontal tape measure around the midpoint, and took two readings to the nearest 0.1 mm. To assess obesity in pregnant women, we calculated the Sum of Skinfold Thickness (SSFT) by taking the average value of biceps, triceps, and subscapular skinfold thickness using Calipers (Holtain, UK) and taking three readings to the nearest 0.2 mm. We measured the mother’s blood pressure (BP) using an automated digital device (Omron Digital BP measuring device) and noted two systolic and diastolic BP readings. Using the Joint National Committee 8 (JNC 8) criteria, blood pressure categorization was performed [45]. Several covariates measured in the cohort found to have significant association with PA during pregnancy in our earlier analysis [46, 47].

### Statistical analysis

The data were checked for assumptions of regression such as normality (outcome variables), multicollinearity, outliers, and linear relationship between independent and dependent variables. After data cleaning and validation, complete data available for exposure, outcome variable and covariates were considered in the final analysis. Data analyses were performed using the Statistical Package for the Social Science (SPSS) version 23.0 (IBM Corp. Released 2015, Armonk, NY) statistical software. PAL is considered an independent variable, whereas gestational age at delivery, the anthropometric parameter measured at delivery, at 14 weeks, and at one year of the child were dependent variables. We assessed the normality of data before applying parametric tests. We used the chi-square test to determine if a difference between observed data and expected data is due to chance, or if it is due to a relationship between the exposure variable (e.g., sedentary behavior) and outcome variables (such as preterm delivery and infant adiposity). We reported frequency and percentage using cross-tabulations for all categorical variables. *P*-value < 0.05 was considered statistically significant, and Fisher exact *p*-values were reported wherever the cell count was less than five. We conducted linear regression to assess the linear relationship between the continuous value of PAL and continuous measurement values of gestational age at delivery and child’s adiposity parameters such as weight, CRL, length, waist and hip circumference, MUAC, head circumference, PI, and SSFT. For adjusted analysis, all other adiposity parameters are considered confounders, and the beta coefficient, 95% CI, and *p*-value were reported. We performed multivariable logistic regression analysis to estimate the adjusted odds ratio between sedentary behavior and preterm delivery, infant adiposity after adjusting for the potential confounders, such as maternal age, SES, gestational age at the time of recruitment, parity, depressive symptoms, social support, GDM, hypertension, and obesity [12, 48, 49]. We presented the odds ratio, 95% confidence interval, and the *p*-value. *P*-values less than 0.05 were considered significantly different.

### Ethical considerations

We obtained ethical approval from the Institutional Ethics Committee (IEC) of the Indian Institute of Public Health, Bengaluru campus (IEC approval no: IIPHHB/TRCIEC/091/2015). We enrolled only those study participants who voluntarily agreed to participate and provided written informed consent. We obtained informed consent from participants after thoroughly explaining the procedures involved. We conducted the investigation in accordance with the latest version of the Declaration of Helsinki [50].

## Results

**Fig. 1:**
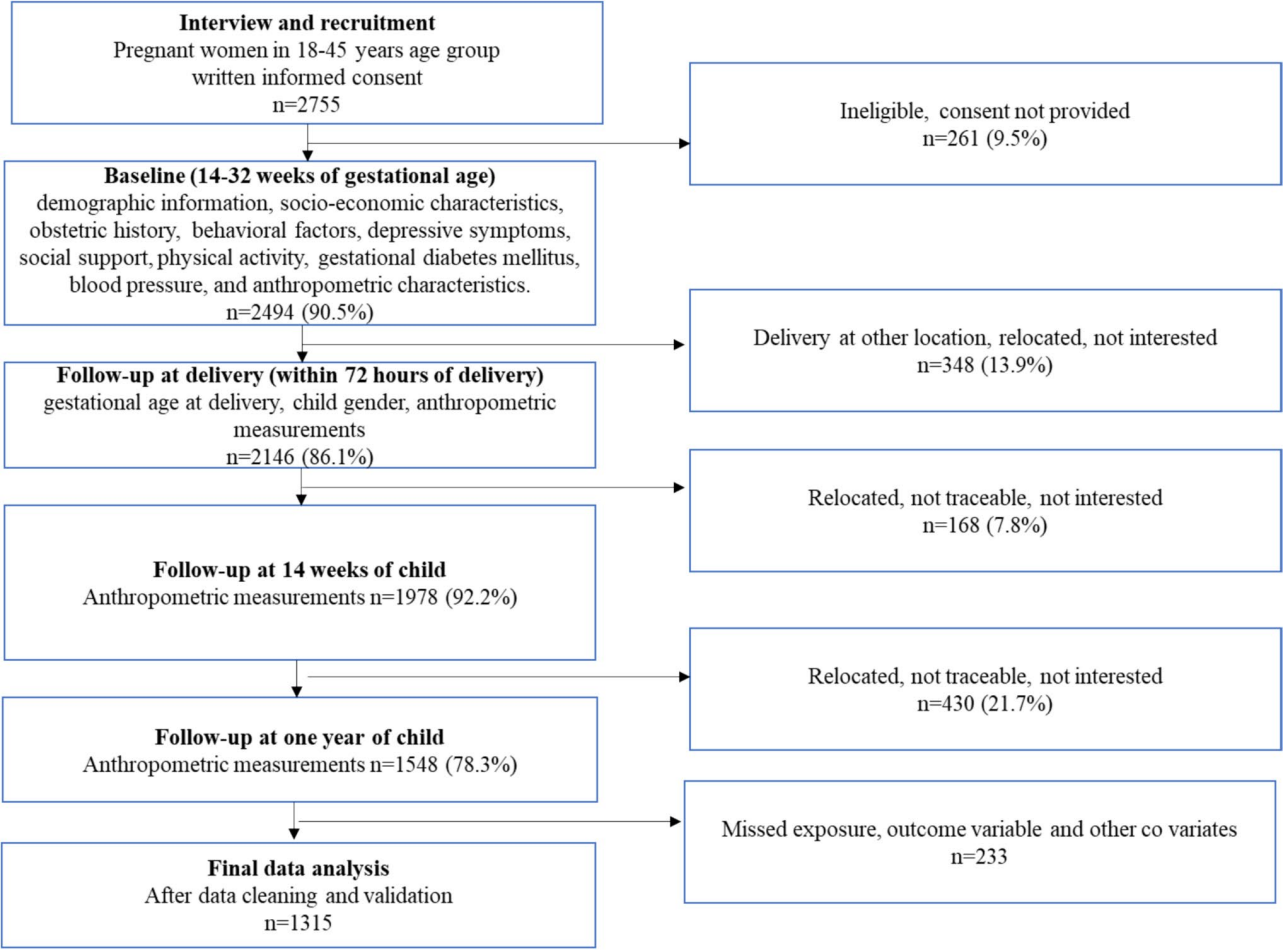
Schematic flow of assessments from recruitment and follow-ups for assessing the association between physical activity during pregnancy with preterm delivery and infant adiposity

We approached 2755 pregnant women and among them, 2494 were eligible and participated in the study. We have completed follow-ups of 2146 soon after delivery, 1978 at 14 weeks of the child, and 1548 at one year of the child. The main reasons for missed follow-up were relocation, not being traceable, and not being interested in further continuing the study. After data cleaning and validation, 1315 were considered in the final analysis of the study (Fig. 1).

Among the pregnant women who were enrolled in this study, 7.1% had sedentary behavior and 10.8% had preterm delivery. About 51% were male babies and high PI was found among 43.4%, 48.9%, and 16.8% of the infants at birth, 14 weeks, and one year age (Table 1).

**Table 1:** Maternal and infant characteristics: categorical and continuous variable distribution, MAASTHI cohort

Assessment	Characteristic	Mean ± SD
Newborn	Gestational age at delivery (week)	38.72 ± 1.51
	Birth weight (kg)	2.77 ± 0.37
	Crown–rump length (CRL) (cm)	31.67 ± 2.62
	Length (cm)	48.12 ± 2.45
	Chest circumference (cm)	31.30 ± 2.04
	Hip circumference (cm)	27.58 ± 2.57
	Sum of skinfold thickness (SSFT) (mm)	13.03 ± 2.45
14 weeks of the child	Weight (kg)	6.19 ± 1.15
	CRL (cm)	40.82 ± 3.67
	Length (cm)	62.48 ± 4.46
	Hip circumference (cm)	38.14 ± 4.16
	SSFT (mm)	21.08 ± 4.45
1 year of the child	Weight (kg)	8.79 ± 1.30
	CRL (cm)	45.41 ± 4.28
	Length (cm)	73.76 ± 4.44
	Hip circumference (cm)	42.87 ± 3.81
	SSFT (mm)	20.22 ± 3.87

	Characteristic	Category	*N* (%)
Pregnant women	Physical activity	Sedentary/light activity	93 (7.1%)
		Moderate activity	1222 (92.9%)
	Gestational age at delivery	Preterm	136 (10.8%)
		Term	1119 (89.2%)
Child	Gender	Male	671 (51.0%)
		Female	644 (49.0%)
	PI at birth	Normal	734 (56.6%)
		High	562 (43.4%)
	PI at 14 weeks	Normal	672 (51.1%)
		High	642 (48.9%)
	PI at 1 year	Normal	792 (83.2%)
		High	160 (16.8%)

N (%) indicates the number and percentage of participants

Mean ± SD represents the mean and standard deviation

Preterm birth: gestational age at birth was less than 37 weeks

Term: gestational age at birth was ≥ 37 weeks

*PI* ponderal index, *SSFT* subscapular skinfold thickness

Among the pregnant women with moderate activity, 48.7% of the newborns had high length, which was significant from women with sedentary behavior. Among the pregnant women who had sedentary behavior, 17.6% had high PI and 9.2% had a higher sum of skinfold thickness at one year of age of their child, which was statistically significant from women with moderate activity (Table 2).

**Table 2:** Distribution of newborn and infant characteristics with the level of physical activity of mothers during pregnancy in the MAASTHI birth cohort

Assessment	Variables	Category	Sedentary/light active	Moderately active	*P*-value
At birth	Delivery gestational age (GA) (weeks)	Preterm	11 (13.3)	125 (10.7)	0.46
		Term	72 (86.7)	1047 (89.3)	
	Weight (kg)	≤ 90 th percentile	117 (94.4)	991 (93.5)	0.63
		> 90 th percentile	7 (5.6)	69 (6.5)	
	Length (cm)	≤ 90 th percentile	55 (63.2)	629 (51.3)	0.03
		> 90 th percentile	32 (36.8)	598 (48.7)	
	Hip circumference (cm)	≤ 90 th percentile	82 (94.3)	1123 (91.4)	0.42
		> 90 th percentile	5 (5.7)	105 (8.6)	
	Ponderal index	normal	69 (79.3)	802 (65.4)	0.08
		High	18 (20.7)	425 (34.6)	
	Subscapular skinfold thickness (SSFT) (mm)	≤ 90 th percentile	80 (92.0)	1120 (91.3)	0.82
		> 90 th percentile	7 (8.0)	107 (8.7)	
	Weight (kg)	≤ 90 th percentile	125 (93.3)	1103 (93.4)	0.96
		> 90 th percentile	9 (6.7)	78 (6.6)	
	Length (cm)	≤ 90 th percentile	34 (39.1)	592 (48.2)	0.09
		> 90 th percentile	53 (60.9)	635 (51.8)	
	Hip circumference (cm)	≤ 90 th percentile	442 (92.9)	443 (92.3)	0.80
		> 90 th percentile	34 (7.1)	37 (7.7)	
	Ponderal index	normal	35 (40.2)	608 (50.1)	0.07
		High	52 (59.8)	605 (49.9)	
	SSFT (mm)	≤ 90 th percentile	79 (90.8)	1111 (90.8)	0.99
		> 90 th percentile	8 (9.2)	113 (9.2)	
1 year	Weight (kg)	≤ 90 th percentile	493 (93.4)	392 (91.6)	0.29
		> 90 th percentile	35 (6.6)	36 (8.4)	
	Length (cm)	≤ 90 th percentile	102 (97.1)	781 (92.0)	0.06
		> 90 th percentile	3 (2.9)	68 (8.0)	
	Hip circumference (cm)	≤ 90 th percentile	442 (92.9)	443 (92.3)	0.80
		> 90 th percentile	34 (7.1)	37 (7.7)	
	Ponderal index	Normal	726 (82.4)	66 (93.0)	0.02
		High	155 (17.6)	5 (7.0)	
	SSFT (mm)	≤ 90 th percentile	476 (90.8)	410 (94.7)	0.02
		> 90 th percentile	48 (9.2)	23 (5.3)	

Percentages are given in parentheses

*p*-values < 0.05 indicate statistical significance between sedentary and moderate activity groups

Preterm birth: gestational age at birth was less than 37 weeks

Term: gestational age at birth was ≥ 37 weeks

*GA* gestational age, *SSFT* subscapular skinfold thickness

Each unit increase in physical activity level (PAL) value is accompanied with an increase in gestational age at delivery (β = 0.007, *p* < 0.001). Moreover, each unit increase in the PAL was found to have a positive effect on the length and decrease in the ponderal index of the newborns. Even at 14-week age of the child, each unit increase in PAL value resulted in a decrease in hip circumference (β = −0.001, *p* = 0.033) and ponderal index (β = −0.06, *p* = 0.04), and at one year age of the child, each unit increase in PAL value, resulted in a decrease in the ponderal index of the child (β = −0.004, *p* = 0.034) (Table 3).

**Table 3:** Linear regression analysis of physical activity level of pregnant women, gestational age at delivery, and anthropometric markers of child adiposity in the MAASTHI birth cohort at birth, 14 weeks, and one year, 2016–2021

Follow-up	Variables	β Coefficient^*^	*p*-value	95% CI
At birth	Delivery gestational age	0.007	** < 0.001**	0.008, 0.005
	Weight (kg)	−0.001	0.93	−0.002, 0.001
	Length (cm)	0.01	**0.02**	−0.001, 0.020
	Hip circumference (cm)	0.001	0.09	−0.001, 0.008
	The sum of skinfold thickness (mm)	−0.003	0.72	−0.005, 0.001
	Ponderal index	−0.007	0.02	−0.009, 0.006
14 weeks	Weight	0.03	0.98	0.01, 0.08
	Length	0.003	0.50	−0.001, 0.006
	Hip circumference	−0.001	**0.03**	−0.003, 0.002
	The sum of skinfold thickness	−0.01	0.07	−0.001, 0.04
	Ponderal index	−0.06	**0.04**	−0.12, −0.002
1 year	Weight	0.001	0.63	−0.001, 0.002
	Length	−0.001	0.13	−0.002, 0.001
	Hip circumference	0.01	0.65	−0.001, 0.04
	The sum of skinfold thickness	−0.002	0.79	−0.003, 0.001
	Ponderal index	−0.004	**0.03**	−0.006, −0.002

*Adjusted for pregnancy gestational age, age of the mother, religion, education, SES, parity, prenatal depression score, social support score, GDM, sum of skinfold thickness of mother, gender of the child

Numbers highlighted in bold represent the significant association between the exposure and outcome variables, such as gestational age at delivery and various anthropometric measurements assessed during each follow-up visit

The odds of having preterm delivery are 1.43 times higher for women with sedentary behavior during pregnancy than for women with moderate activity (OR = 1.43, CI 1.15, 3.77 *p* = 0.041). Also, women who had sedentary behavior had higher odds for hip circumference at the 14-week age of the child (OR = 2.53, CI 1.001, 3.72, *p* = 0.007) and ponderal index (OR = 1.65, CI 1.05, 2.59, *P* = 0.031). Similarly, the odds of having obesity among children at 1 year of age is 1.53 times and 1.64 times higher as measured through the sum of skinfold thickness and ponderal index, respectively, than for women with moderate activity (Table 4).

**Table 4:** Association of sedentary behavior during pregnancy with preterm delivery and child adiposity outcomes at birth, 14 weeks, and one year of the child in the MAASTHI cohort: logistic regression analysis, 2016–2021

Follow-up age	Variable	OR*	*p*-value	95% Confidence interval (CI)
At birth	Preterm delivery	1.43	**0.04**	1.15, 3.77
	Overweight	1.009	0.97	0.64, 1.60
	Length	1.63	0.06	1.04, 2.56
	Hip circumference	1.53	0.36	0.60, 3.86
	Sum of skinfold thickness	2.64	0.18	1.00, 3.84
	Higher ponderal index	1.83	**0.03**	1.03, 2.94
14 weeks	Weight	1.05	0.84	0.67, 1.65
	Length	1.23	0.16	1.00, 1.99
	Hip circumference	2.53	**0.007**	1.48, 3.72
	Sum of skinfold thickness	2.62	0.89	1.15, 4.83
	Higher ponderal index	1.64	**0.03**	1.05, 2.59
One year	Weight	1.20	0.47	0.73, 1.99
	Length	1.32	0.28	0.80, 2.19
	Hip circumference	1.84	0.24	0.65, 5.19
	Sum of skinfold thickness	1.53	**0.02**	1.32, 2.02
	Higher ponderal index	1.64	**0.03**	1.31, 2.95

*Adjusted for pregnancy gestational age, maternal age, religion, education, socioeconomic status (SES), parity, prenatal depression score, social support score, gestational diabetes mellitus (GDM), maternal sum of skinfold thickness, and child gender

Numbers highlighted in bold represent the significant association between the exposure and outcome variables, such as gestational age at delivery and various anthropometric measurements assessed during each follow-up visit

## Discussion

In this study within the MAASTHI birth cohort, we assessed whether sedentary behavior during pregnancy was associated with preterm delivery and adiposity markers in newborns and infants. We showed that pregnant women with sedentary behavior had higher odds of preterm birth and adiposity in infants as indicated by increased sum of skinfold thickness, hip circumference, and ponderal index both at 14 weeks and at one-year follow-up.

Asian countries report lower physical activity levels during pregnancy, with a gradual decline over time [5]. The study results are consistent with existing research that links maternal PA with various neonatal outcomes. Women who engaged in mid-to-late term physical activity gave birth to infants with less fat mass than those who had little to no physical activity throughout pregnancy [51]. A vigorous form of PA in the third trimester of pregnancy was associated with lower child fat mass at the one year age of the child [3]. Meta-analysis of cohort studies found that PA during late pregnancy is consistently associated with a modestly lower risk of large for gestational age (LGA) babies and newborns with macrosomia [52].

Our study findings align with existing literature indicating that there is no significant association between maternal PA and the birth weight of newborns but had an association with the ponderal index of the newborn. This consistency in results reinforces the understanding that maternal PA might not be a determining factor for neonatal birth weight as alone, [53] but when weight and length both were considered through ponderal index at birth, PA has association with it. These findings convey that both weight and length are crucial in determining adiposity of the newborn. However, the role of PA in relation to preterm birth is complex; our study observed that moderate levels of PA during pregnancy were associated with lower odds of preterm birth compared to sedentary behavior [54]. This is consistent with findings from other studies [55]. Also, adequate PA during third trimester of pregnancy offers protection to preterm birth [56].

It is important to increase PA to optimal levels and maintain it throughout pregnancy. However, determining the optimal level to be achieved during pregnancy can be difficult to understand practically, as each trimester requires different energy needs and expenditures. Moreover, knowledge of the effects of maternal PA on pregnancy and child health outcomes may encourage pregnant women to be more physically active during pregnancy.

The Global Action Plan on PA emphasizes the importance of integrating PA into the daily part of life of individuals across the life course [57]. The WHO 2020 PA guidelines stress the importance of the life course approach to reduce sedentary behaviors [58]. PA during pregnancy has not been fully incorporated into life course research, despite mounting evidence for its importance and potential insights using theoretical frameworks [59]. A recent review highlighted the benefits of incorporating life course epidemiology into PA research [60].

Sedentary behavior is a growing public health problem in LMICs, and promoting PA during pregnancy could have significant benefits for maternal and perinatal health outcomes. The relevance of our study extends beyond Indian urban settings to other LMICs where the burden of adverse maternal and fetal outcomes is high. Earlier systematic reviews and meta-analysis urged the need for future research to assess the influence of maternal physical activity on children by conducting longer follow-up to at least one year of children [61] and the present study adds to such evidence through setting a large transgenerational cohort and following up both mother and child at regular intervals during one year postpartum. Earlier established evidence suggests that it is feasible to set up a birth cohort study at public health facilities, [62] and it also paved the way to explore transgenerational factors associated with infant adiposity.

The current findings of the study show a significant association of the sedentary behavior of pregnant women with preterm delivery and infant adiposity needs further assessment. Future studies should focus on the studies that aim to further explore the relationship between PA during each trimester of pregnancy on child health outcomes using cohort studies and also physiological mechanisms which results in such association. It is crucial to conduct studies that objectively measure the level and intensity of PA using wearable devices, as they can accurately track the duration of each activity. Awareness programs to improve the knowledge and practice of PA among women in the childbearing age group must be strengthened. Strategies need to be made to promote and enhance PA among pregnant mothers.

### Strengths and limitations of the study

Our study was conducted by using a large sample size at public hospitals, where the majority of pregnant mothers seek health care, and demonstrated the potential benefits of PA during pregnancy in an urban Indian population. This study used validated questionnaires and biochemical tests were conducted at accredited laboratory facilities, taking internal and external quality check measures. Moreover, research staff were trained and certified for data collection and performing anthropometry measurements.

Nutritional factors, maternal dietary practices, socio-cultural customs, pre-pregnancy health status, and gestational weight gain might have confounded the association and the study could not assess these confounding factors. Questionnaires used to assess the level of PA were subjective data, and there could be recall bias, and there might be a chance of over-reporting due to giving socially desirable answers. To minimize it, we sustained the anonymity and privacy of the gathered data. This study has not found pregnant women with vigorous levels of PA category, and hence, only sedentary and moderate-level PA were considered for analysis. Since the PA data were collected during the recruitment of the pregnant mother, i.e., in the second trimester and early third trimester, this study could not assess the increased or decreased level of PA in each trimester and its influence on infant adiposity. Study findings could not be generalized beyond the urban Indian setting since rural and semi-urban areas were not included. Also, several cultural, economic, and infrastructural differences and contextual factors could influence generalizability and outcome in different settings.

## Conclusion

Our study highlights that sedentary behavior during pregnancy is associated with preterm births and adiposity markers in infants. These findings suggest the importance of moderate levels of physical activity during prenatal care, which were associated with lowering the chance of preterm birth and infant adiposity. Therefore, health care workers have a crucial role in assessment and provide awareness about optimal level of activity to women. Further research, especially in low and middle-income countries, is needed to evaluate the effectiveness of PA interventions among pregnant women to prevent infant adiposity.

## Data Availability

The datasets generated for this study are available on request to the corresponding author.
